# Influence of periodontal disease on systemic disease: inversion of a paradigm: a review

**Published:** 2013-06-25

**Authors:** M Bansal, S Rastogi, NS Vineeth

**Affiliations:** * Department of Periodontology, Institute of Dental Studies and Technologies, CCS University, Modinagar, Uttar Pradesh, India; ** Department of Oral and Maxillofacial Surgery and Oral Implantology, Institute of Technology and Sciences- Centre for Dental Studies and Research, Murad Nagar, Ghaziabad, India; *** Dept of Oral and Maxillofacial Surgery, Shree Bankey Bihari Dental College, Ghaziabad

**Keywords:** Periodontal diseases, oral infection, systemic health

## Abstract

Medicine and dentistry interface at many levels. For example, the focal infection theory, popular at the outset of the 1900s, suggested that systemic ailments could be traced to dental infections, which, in those days, were common, chronic, and often untreated. With the advent of modern dental and medical treatment, particularly antibiotics, this relationship was largely forgotten. Until recently, the discovery of relationships between periodontal disease and heart ailments, maternal oral health and prematurity of offspring, bidirectional interrelationships between diabetes and periodontal diseases, relationship of oral infections and chronic respiratory diseases and relationship between skeletal and oral bone mineral density, has brought a shift in the perspective. Research is now focused on the potential impact of periodontal diseases on systemic health. Thus, the impact of oral infection in systemic health defined a novel branch in Periodontology termed Periodontal medicine.

## Introduction

The organization of health profession into specialties and sub specialties according to body organs and systems is often more pragmatic than scientific. The human organism is a single unit composed of a seemingly infinite number of biologic processes so intertwined that abnormalities of almost any of its parts or processes have profound effects in multiple body areas. The link between the oral cavity and general health is similar and it can be stated very correctly, “The mouth is the window to your body’s health". It can show signs of illnesses, general infections and nutritional deficiencies [**1**].

### The turning of tide in dentistry

Oral sepsis was first introduced in the medical literature in a report entitled “Oral sepsis as a cause of disease" by Wiliam Hunter in 1900 [**2**]. This was superseded by focal infection introduced by Frank Billings in 1911 [**3**]. He defined it as a circumscribed area of tissue infected with pathogenic organism. Focal infection has continued to be explored as a possible cause or exacerbating factor of some systemic conditions, but is now being evaluated on a scientific basis [**4**]. The concept of focal infection has always been recognized as being potentially causal in bacterial endocarditis. Most recently, intense attention focused on oral sepsis and its relation to the causology of conditions such as cardiovascular diseases, diabetes, respiratory disorders, osteoporosis and adverse pregnancy outcomes. Thus, the impact of oral infection on systemic health further defined the new branch of periodontology termed as “Periodontal medicine".

### Role of oral environment in cardiovascular diseases

**Fig. 1:**
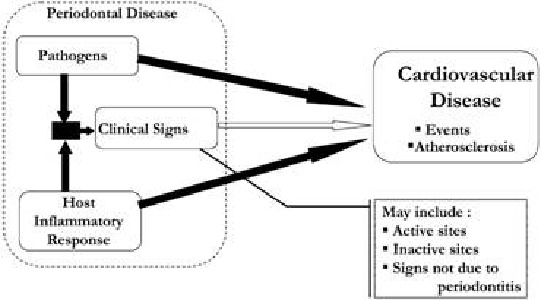
Flow chart showing the link between periodontal disease and cardiovascular diseases

**Fig. 2:**
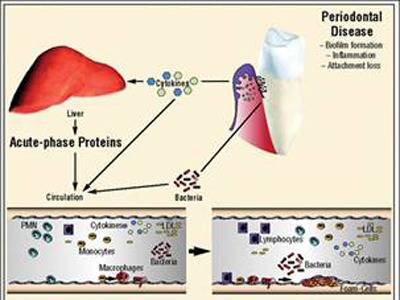
Acute phase response

**Fig. 3:**
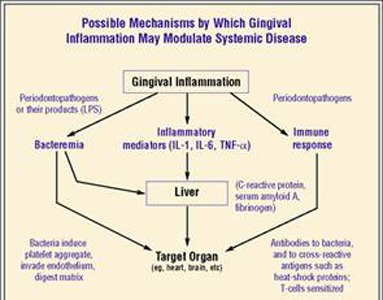
Release of cytokines

The recent data linking cardiovascular disease and periodontitis can elicit a systemic inflammatory response by activating the hepatic acute phase response (**Fig. 1**), this occurs presumably as a consequence of the systemic appearance of transient and recurrent bacteraemia of oral origin which has been a long recognized characteristic of periodontal infections. Cross sectional evidence indicates that periodontitis elicits a mild elevation in the markers of the acute phase response including C-reactive protein, haptoglobin, alpha 1 antitrypsin and fibrinogen (**Fig. 2**). The acute phase proteins is triggered by blood borne oral lipopolysaccharides and oral bacteria which elicit the release of cytokines IL-6 and TNF-α (**Fig. 3**). Recent data generated in the cardiovascular research have demonstrated that mild elevation in C-reactive protein appears to be associated with an increased risk for both incident myocardial infarction and new diagnosis of peripheral artery disease in apparently healthy individuals. [5,6].

### Role of oral environment in the initiation and progression of diabetes

**Fig. 4:**
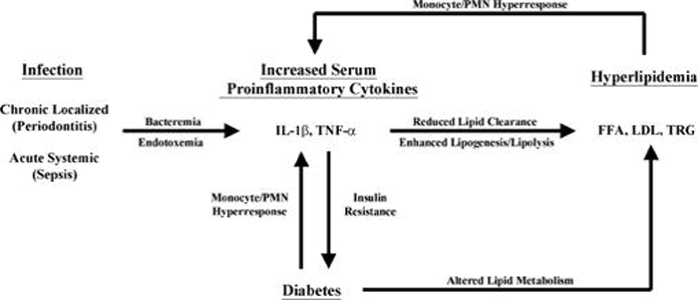
Exacerbation of progression of systemic disease by oral infection

Periodontal infection represents a complication that may be involved in altering the systemic physiology in diabetic patients. Since periodontitis can be more than just a localized oral infection, the effects have been hypothesized to be far reaching [**7**]. Severe chronic forms of this disease can result in systemic response to the bacteria and bacterial products that are disseminated due to the breakdown of periodontal apparatus. The interrelationships between diabetes and periodontal disease provide an example of systemic disease predisposing to oral infection and once that infection is established, the oral infection exacerbates the progression of systemic disease (**Fig. 4**). 

In addition, it is also possible for the oral infection to serve as a metabolic stressor that may exacerbate the systemic disease. Bacterial products such as endotoxin or LPS also play a role in propagating an inflammatory response in the host through the Toll like receptors (TLR’s) and thus can induce an inflammatory cascade [**8**]. These receptors play an important role in the innate immune response. Particularly, the initial interaction between the infecting microorganisms, such as P. gingivalis and phagocytic cells of monocytic lineage [**9**].

 A number of reviews and studies have proposed mechanisms to explain the relationship including:

 • Microvascular disease

 • Changes in components of GCF

 • Changes in collagen metabolism

 • Altered host response

 • Altered subgingival flora

 • Genetic predisposition

 • Non enzymatic glycation

 As a consequence of an infectious challenge, cytokine production could potentially contribute to insulin resistance in a number of ways including [**10**]:

 • Modification of insulin receptor substrate-1 by serine phosphorylation

 • Alteration of adipocyte function with increased production of free fatty acids

 • Diminution of endothelial NO production

 In fact, the cytokine-induced mechanism has been suggested to participate in the β cell damage or burn out seen in animal obesity models of type-2 diabetes. Based on the potential mechanistic interrelationships between diabetes and periodontal diseases, there are two major areas for potential therapeutic interventions. The putative cyclical relationship may be interrupted by reducing serum cytokine or lipid levels. Reduction of serum cytokine levels requires the use of drugs, monoclonal antibodies directed against IL-1β/TNF-α or receptor antagonist targeted specifically to the IL-1β or TNF- α receptors [**11**].

### Role of oral environment in respiratory diseases

**Fig. 5:**
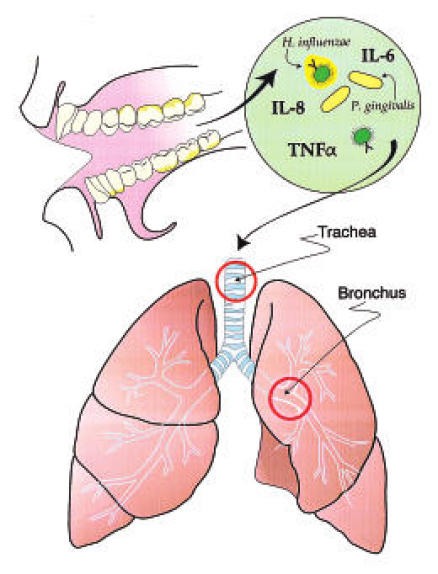
Participation of oral bacteria in the pathogenesis of respiratory disease

A number of recent microbiologic and epidemiologic studies have suggested a relationship between poor oral health and respiratory disease especially in high-risk subjects. Several mechanisms can be envisioned to explain how oral bacteria can participate in pathogenesis of respiratory infection (**Fig. 5**).

#### Pathogenesis

**Fig. 6:**
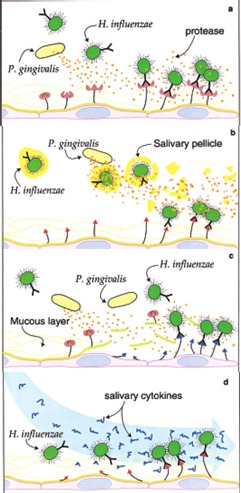
Periodontal disease associated enzymes in saliva may modify mucosal surfaces to promote adhesion and colonization by respiratory pathogens

 Although the colonization of the digestive tract has been suggested to be a source of hospital acquired pneumonia, recent evidence now supports the oropharyngeal region as the likely source of bacteria [**12**]. Several mechanisms can be envisioned to explain how oral bacteria can participate in the pathogenesis of respiratory infection:

 • Aspiration of oral pathogens (P. gingivalis, A actinomycetemcomitans)

 • Periodontal disease associated enzymes in saliva may modify mucosal surfaces to promote adhesion and colonization by respiratory pathogens

 • Periodontal disease associated enzymes may destroy salivary pellicles on pathogenic bacteria

 • Cytokines originating from periodontal tissues may alter respiratory epithelium to promote infection by respiratory pathogens [**13**] (**Fig. 6**).

### Oral environment and adverse pregnancy outcomes

The international definition of low birth weight adopted by the 29th world health assembly in 1976 is a birth weight less than 2500 grams [**14**]. The normal gestation for human full term is of 40 weeks. Preterm or premature birth is usually defined as a gestational age of less than 37 weeks [**15**].

**Fig. 7:**
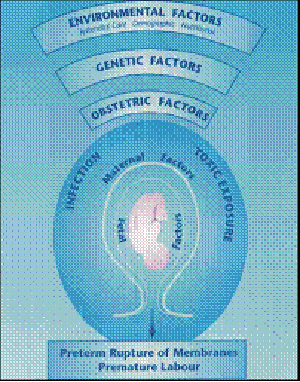
Factors responsible for premature rupture of membranes

 Galloway [**16**] first suggested in 1931 that periodontal disease has more than just an association but actually contributes to a low birth weight. Later, in 1996, Offenbacher et al. [**17**] suggested that periodontal infections serve as chronic reservoirs of LPS, which could target the placental membranes via the blood stream and LPS has been shown to elicit the production of IL-1β and PGE2 by the chorio-amnionic and trophoblastic cells, a process often associated with pre term parturition (**Fig. 7**).

**Fig. 8:**
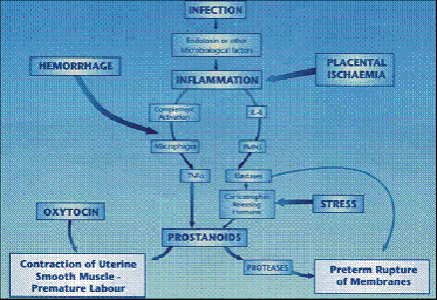
Pathogenesis of periodontal infection & premature rupture of membranes

 Jeffcoat et al. [**18**] reported that with their existing case control studies and prospective and uncontrolled intervention studies there was evidence suggesting that pre existing periodontal disease in the second trimester of pregnancy increases the risk of a pre term birth. Furthermore, animal experiments in which pregnant hamsters were challenged with P. gingivalis and gram-negative bacteria frequently associated with periodontitis have shown significant association between the increasing levels of PGE2 and TNF-α and fetal growth retardation. Such findings could suggest that the infection with P. gingivalis may affect human pregnancy outcome [**19**] (**Fig. 8**).

### Role of oral environment on osteoporosis

**Fig. 9:**
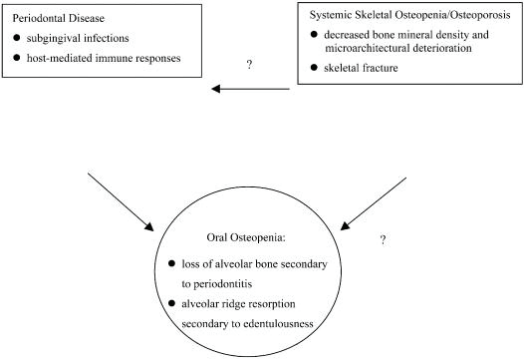
Pathogenesis of periodontal infection & osteoporosis

Osteoporosis is a skeletal disorder characterized by compromised bone strength predisposing to increased risk of fracture with bone strength determined by the low bone density and bone quality [**20**]. Periodontitis results from bacteria that produce factors that cause loss of collagenous support of the tooth, as well as the loss of the alveolar bone. Although the periodontal disease has historically been thought of to be result of an infectious process, others have suggested that the periodontal disease may be an early manifestation of generalized osteopenia [**21**] (**Fig. 9**).

 Mechanisms by which osteoporosis or systemic bone loss may be associated with periodontal attachment loss, loss of alveolar bone height and tooth loss continues to be explored. Potential mechanisms have been proposed. However, still, the question whether the individuals with oral osteopenia are at risk for systemic osteopenia and osteoporosis is without conclusive answers. This is obviously an important clinical question and should be addressed since dentists could provide an “early warning" awareness of existing osteoporosis risk [**22**].

## Conclusions

Periodontics is entering a new era. Research now suggests that far from being just an oral malady, periodontal diseases and oral infections have been linked with systemic diseases and conditions. Understanding this co-relation is a crucial step for both dental and medical professionals in determining the best approach to patient care. Therefore, the aim of this paper was to forge a coalition between the clinicians who treat the oral disease and those who treat systemic conditions, and, to provide a forum for new emerging data and to identify directions for future research. Acknowledgement: None Sources of Funding: None Disclosures: None
